# Mathematical expansion and clinical application of chronic kidney disease stage as vector field

**DOI:** 10.1371/journal.pone.0297389

**Published:** 2024-03-13

**Authors:** Eiichiro Kanda, Bogdan I. Epureanu, Taiji Adachi, Tamaki Sasaki, Naoki Kashihara

**Affiliations:** 1 Medical Science, Kawasaki Medical School, Kurashiki, Okayama, Japan; 2 College of Engineering, University of Michigan, Ann Arbor, Michigan, United states of America; 3 Institute for Life and Medical Sciences, Kyoto University, Sakyo, Kyoto, Japan; 4 Department of Nephrology and Hypertension, Kawasaki Medical School, Kurashiki, Okayama, Japan; 5 Kawasaki Geriatric Medical Center, Kita, Okayama, Japan; University of Glasgow, UNITED KINGDOM

## Abstract

There are cases in which CKD progression is difficult to evaluate, because the changes in estimated glomerular filtration rate (eGFR) and proteinuria sometimes show opposite directions as CKD progresses. Indices and models that enable the easy and accurate risk prediction of end-stage-kidney disease (ESKD) are indispensable to CKD therapy. In this study, we investigated whether a CKD stage coordinate transformed into a vector field (CKD potential model) accurately predicts ESKD risk. Meta-analysis of large-scale cohort studies of CKD patients in PubMed was conducted to develop the model. The distance from CKD stage G2 A1 to a patient’s data on eGFR and proteinuria was defined as *r*. We developed the CKD potential model on the basis of the data from the meta-analysis of three previous cohort studies: ESKD risk = exp(*r*). Then, the model was validated using data from a cohort study of CKD patients in Japan followed up for three years (n = 1,564). Moreover, the directional derivative of the model was developed as an index of CKD progression velocity. For ESKD prediction in three years, areas under the receiver operating characteristic curves (AUCs) were adjusted for baseline characteristics. Cox proportional hazards models with spline terms showed the exponential association between *r* and ESKD risk (*p*<0.0001). The CKD potential model more accurately predicted ESKD with an adjusted AUC of 0.81 (95% CI 0.76, 0.87) than eGFR (*p*<0.0001). Moreover, the directional derivative of the model showed a larger adjusted AUC for the prediction of ESKD than the percent eGFR change and eGFR slope (*p*<0.0001). Then, a chart of the transformed CKD stage was developed for implementation in clinical settings. This study indicated that the transformed CKD stage as a vector field enables the easy and accurate estimation of ESKD risk and CKD progression and suggested that vector analysis is a useful tool for clinical studies of CKD and its related diseases.

## Introduction

Chronic kidney disease (CKD) patients have high risks of end-stage kidney disease (ESKD), cardiovascular disease (CVD), and death [1–3]. The accurate predictions of renal function and life expectancy are useful for diagnosing CKD patients with high risk of ESKD and determining appropriate therapeutic strategies for them [4,5].

In recent years, the National Kidney Foundation and the US Food and Drug Administration proposed percent (%) changes in estimated glomerular filtration rate (eGFR) and proteinuria as well as the eGFR slope as surrogate endpoints for ESKD [6–9]. These indices are effective in clinical studies. However, when decreases in eGFR and proteinuria are observed in a patient, it is difficult to decide whether the therapy is effective or not, because it is unclear whether either eGFR or proteinuria is more important. Previous indices for CKD progression treat eGFR and proteinuria as independent variables and have similar problems [10,11]. Indices directly indicating ESKD risks would be useful for evaluating CKD progression at the individual level. Thus, we previously created an AI model to predict the progression of CKD and presented it as a web system [12]. However, there were difficulties in using the AI system in clinical settings, such as not knowing how the AI analyzes CKD and business-related problems. Therefore, we needed an indicator that humans can understand and calculate easily.

CKD severity classification is a tool for patient education and screening for high-risk patients [4]. It has interesting characteristics: the CKD stages with the same levels of outcome risks concentrically exist around G2 A1 [4]. Moreover, the risk levels exponentially increase with increasing distance from CKD stage G2 A1. A similar tendency is observed in cohort studies [13–17].

If CKD stage can be treated as a coordinate system, sophisticated mathematical models of CKD can be developed. Vector analysis is a method using vector fields with calculus and is often used in various fields of medicine such as neuroscience and machine learning [18]. Although vector fields are difficult to simply apply to CKD, if the relationship between CKD patient data and outcome risks can be treated as a type of potential, changes in the eGFR and in proteinuria of a patient can be mathematically analyzed as a vector.

In this study, assuming that CKD stage is a vector field and considering ESKD risk as a potential, we aimed to develop a CKD-stage vector field on the basis of the data from the meta-analysis of previous large-scale cohort studies and validate its ESKD risk prediction using Kawasaki CKD cohort study data. Then, we developed a chart of the CKD-stage vector field for application in clinical settings. Moreover, a gradient of the CKD-stage vector field was evaluated for its accuracy to predict CKD progression velocity.

## Results

### Baseline characteristics and distance from the origin

Patients were categorized into six groups on the basis of the distance from the origin, *r* (S1 Table). The greater the distance *r* became, the more eGFR decreased and urinary protein-to-creatinine ratio (UPCR) increased, and more patients tended to be in CKD stages G5 and A3. Various symptoms often appearing with CKD progression tended to be observed with increasing *r*, such as decreased albumin, calcium, higher potassium, and hemoglobin levels, and increased phosphorus, and uric acid levels. Moreover, more patients with ESKD were observed in Groups 5 to 6 than in other groups (S2 Table).

#### CKD-stage vector field

**Fig 1:**
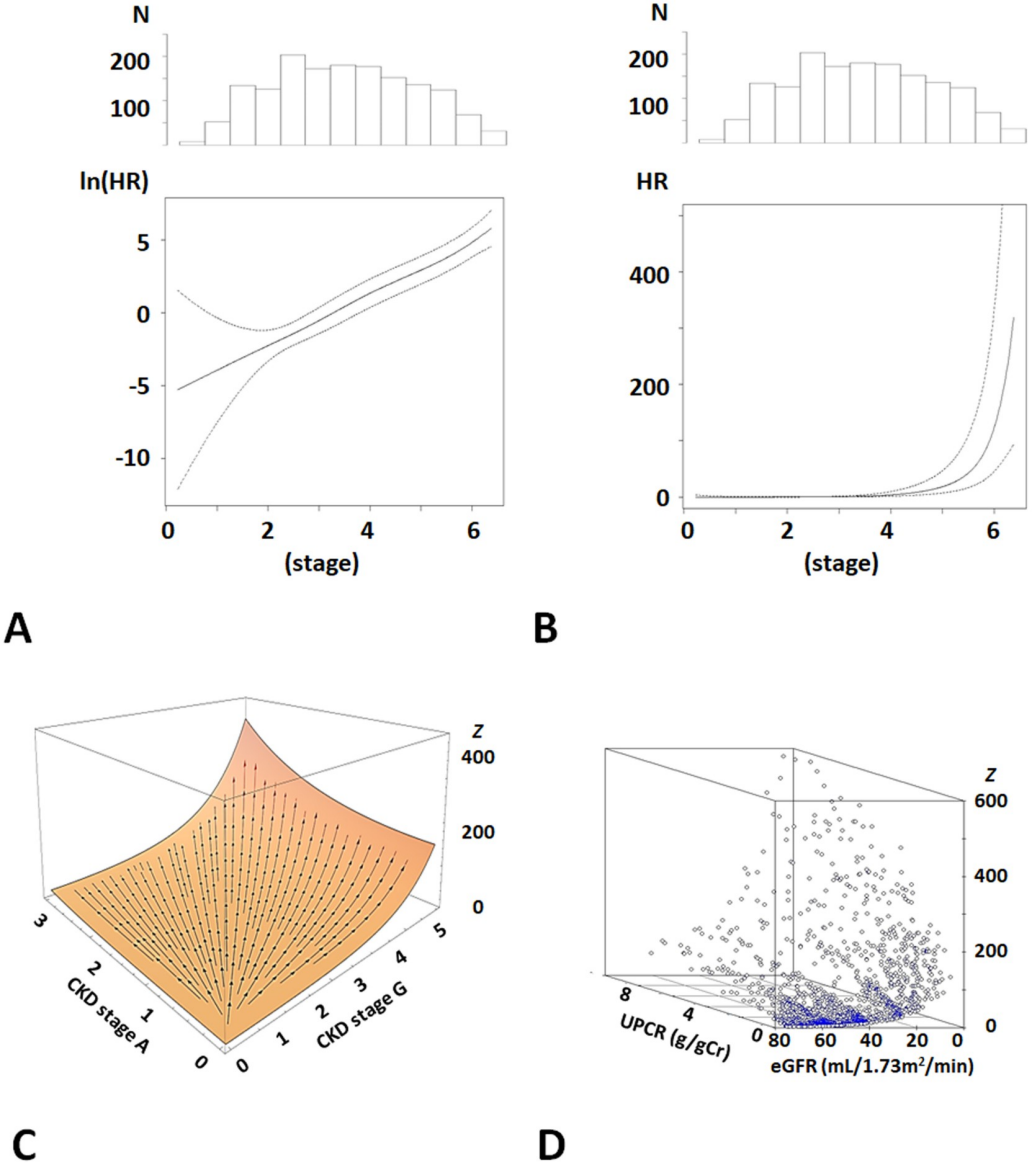
Relationship between CKD-stage vector field and ESKD risk. **A, B.** Histograms of *r*. The ln(HR) and HR of ESKD are shown below the histograms in A and B respectively. A and B show the exponential relationship between *r* and ESKD risk. Ln(HR) and HR were adjusted for the baseline characteristics (reference Methods). **C.** Plane of CKD-stage vector field showing each vector to the direction of highest ESKD risk of each patient. **D.** Distribution of *z* for patients. They are plotted on the plane in C. Abbreviations: HR, hazard ratio adjusted for baseline characteristics; CKD, chronic kidney disease; ESKD, end-stage kidney disease.

*r* was found to be linearly associated with the ln(hazard ratio, HR) of ESKD risk (*p*<0.0001), and the exponential function was considered to be appropriate for the CKD-stage vector field (*p*<0.0001) (Fig 1A and 1B). Meta-analysis of the three cohort studies showed a coefficient *C* of 1.02 (95%CI 0.85, 1.20). From this result, for the CKD-stage vector field, the following was obtained (Fig 1C).

$$z=\text{exp}\left(r\right)$$ (1)

95% CI of *z* was estimated from exp(0.85*r) to exp(1.20*r).

Patients’ data were distributed on the surface of *z* (Fig 1D). *z* increased with *r* (S1 Table) and was associated with eGFR (*ρ* = −0.81, *p*<0.0001) and UPCR (*ρ* = 0.83, *p*<0.0001) (S1 Fig).

**Fig 2:**
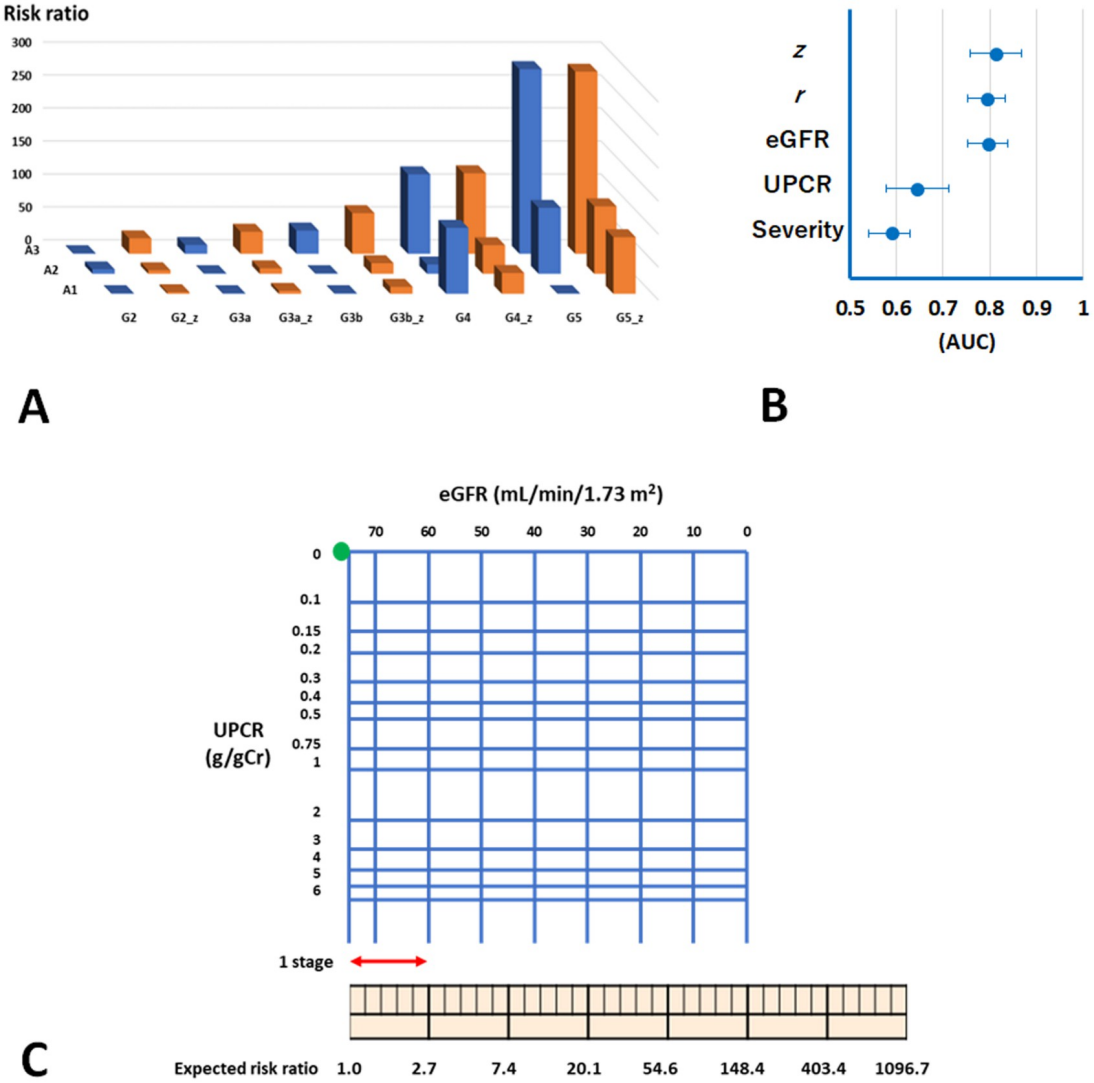
Estimation of ESKD risk on the basis of CKD-stage vector field. **A.** The expected ESKD risk estimated using *z* (brown bars) shows a similar trend to the risk ratio of ESKD referenced to CKD stage G2 A1 in the cohort study (blue bars). **B.** AUCs for the prediction of ESKD. AUCs of eGFR and UPCR were adjusted for the baseline characteristics with UPCR and eGFR, respectively. Those of *z*, *r*, and Severity were adjusted for the baseline characteristics. **C.** Logarithmic chart of transformed eGFR and UPCR. UPCR is shown in the logarithmic axis. This chart is used as follows: (1) Plot a patient’s data on eGFR and UPCR as a dot. (2) Measure the length from the origin to the patient’s dot. Set the length of 1 stage = 1 cm. (3) Read the scale using a ruler or calculate exp(the length). The value shows the risk ratio of ESKD with reference to the origin. Abbreviations: eGFR, estimated glomerular filtration rate; UPCR, urinary protein-to-creatinine ratio; Severity, CKD-severity classification; AUC, areas under the receiver operating characteristic curve.

The risk ratio of ESKD and the ESKD risk estimated using *z* at various CKD stages are shown in Fig 2A. The risk ratio and *z*-estimated risk showed a strong relationship (*ρ* = 0.74, *p* = 0.0014).

Area under the receiver operating characteristic (ROC) curves (AUCs) for the prediction of ESKD within three years, which were adjusted for baseline characteristics, for *z* and other indices are shown in Fig 2B. The AUC of *z* was 0.81 (95% CI 0.76, 0.87) and larger than that of eGFR, 0.79 (95% CI 0.75, 0.84) (*p*<0.0001). The AUCs of *r*, UPCR, and CKD-severity classification were 0.79 (95% CI 0.75, 0.83), 0.64 (95% CI 0.58, 0.71), and 0.59 (95% CI 0.54, 0.63), respectively. These results show that *r* was associated with ESKD risk and that *z* well predicted the risk.

Then, a logarithmic chart of eGFR and UPCR was developed and used with a ruler to easily estimate a patient’s ESKD risk (Fig 2C). It is used as follows: (1) plot a patient’s data as a dot, (2) measure the length from the origin to the patient’s dot, and (3) determine from the reading risk using the ruler. Moreover, the change in ESKD risk as risk ratio between the previous and present medical examinations can be estimated.

#### Indices of CKD progression

The directional derivative, the inner product, and Cos*θ* were higher in Groups 5 and 6 than in other groups (S3 Table). It was shown that CKD progressed at an accelerated speed with the distance from the origin. The negative signs of the directional derivative, the inner product, and Cos*θ* were mainly observed in Group 1, indicating that the possibility of the improvement of kidney function was low in Groups 4 to 6.

**Fig 3:**
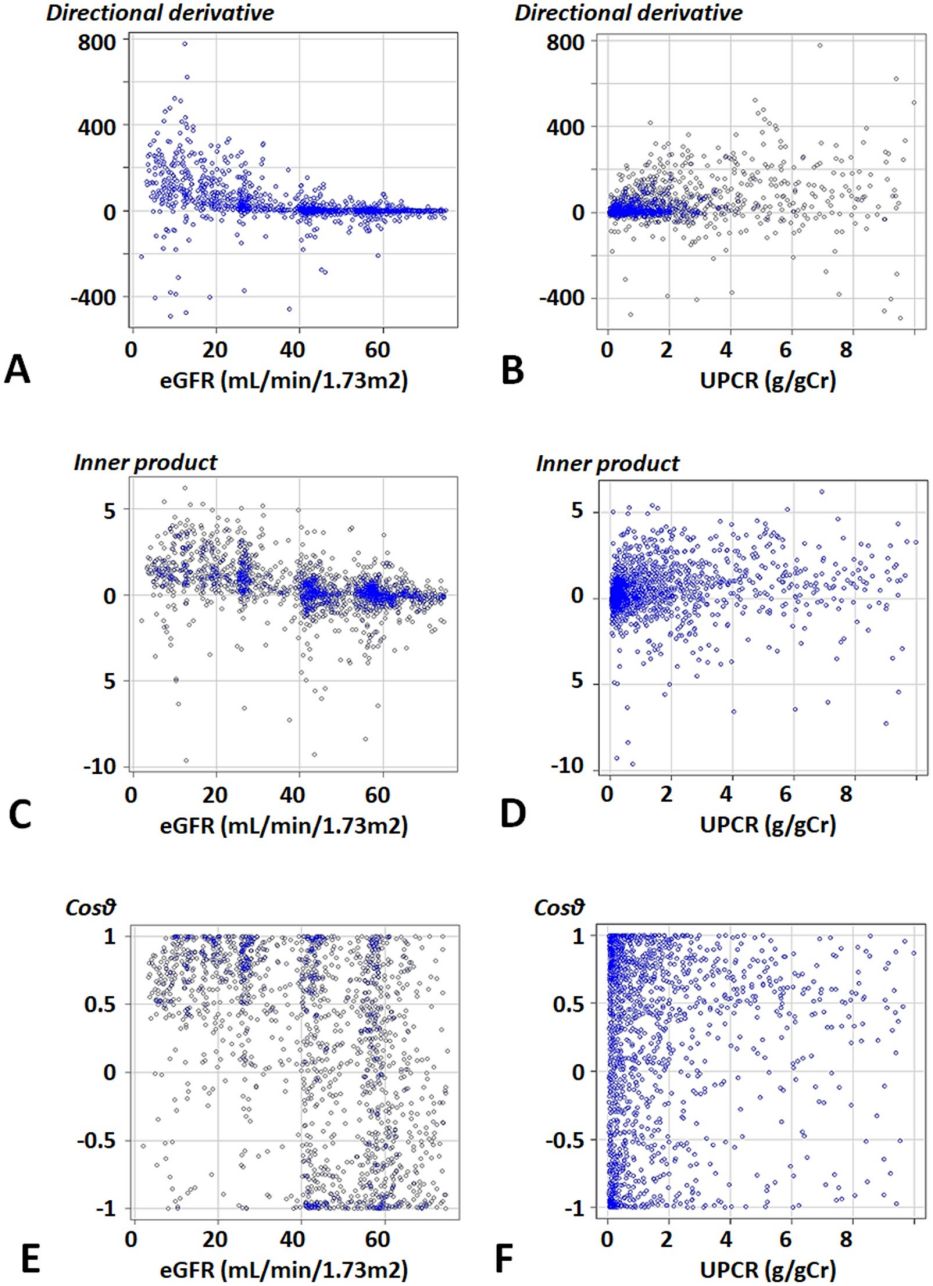
Distributions of indices of CKD progression. **A, B.** Directional derivative. **C, D.** Inner product. **E, F.** Cos*θ*. Abbreviations: eGFR, estimated glomerular filtration rate; UPCR, urinary protein-to-creatinine ratio.

Fig 3 shows the distributions of the indices, eGFR, and UPCR. Positive values of the directional derivative and the inner product were observed in patients with eGFR of less than 40 mL/min/1.73 m^2^ (Fig 3A and 3C). Negative values of Cos*θ* were rarely observed in patients with eGFRs of less than 40 mL/min/1.73 m^2^ (Fig 3E). Moreover, CKD rapidly progressed in patients with UPCRs of 2 g/gCr or more (Fig 3B and 3D). Cos*θ* also indicated that there were only a few patients with UPCRs of less than 2 g/gCr, whose ESKD risk tended to decrease (Fig 3F). Therefore, it was suggested that the effect of CKD was significant on eGFRs of less than 40 mL/min/1.73 m^2^ or UPCRs of 2 g/gCr or more.

#### CKD progression and ESKD risk

**Fig 4:**
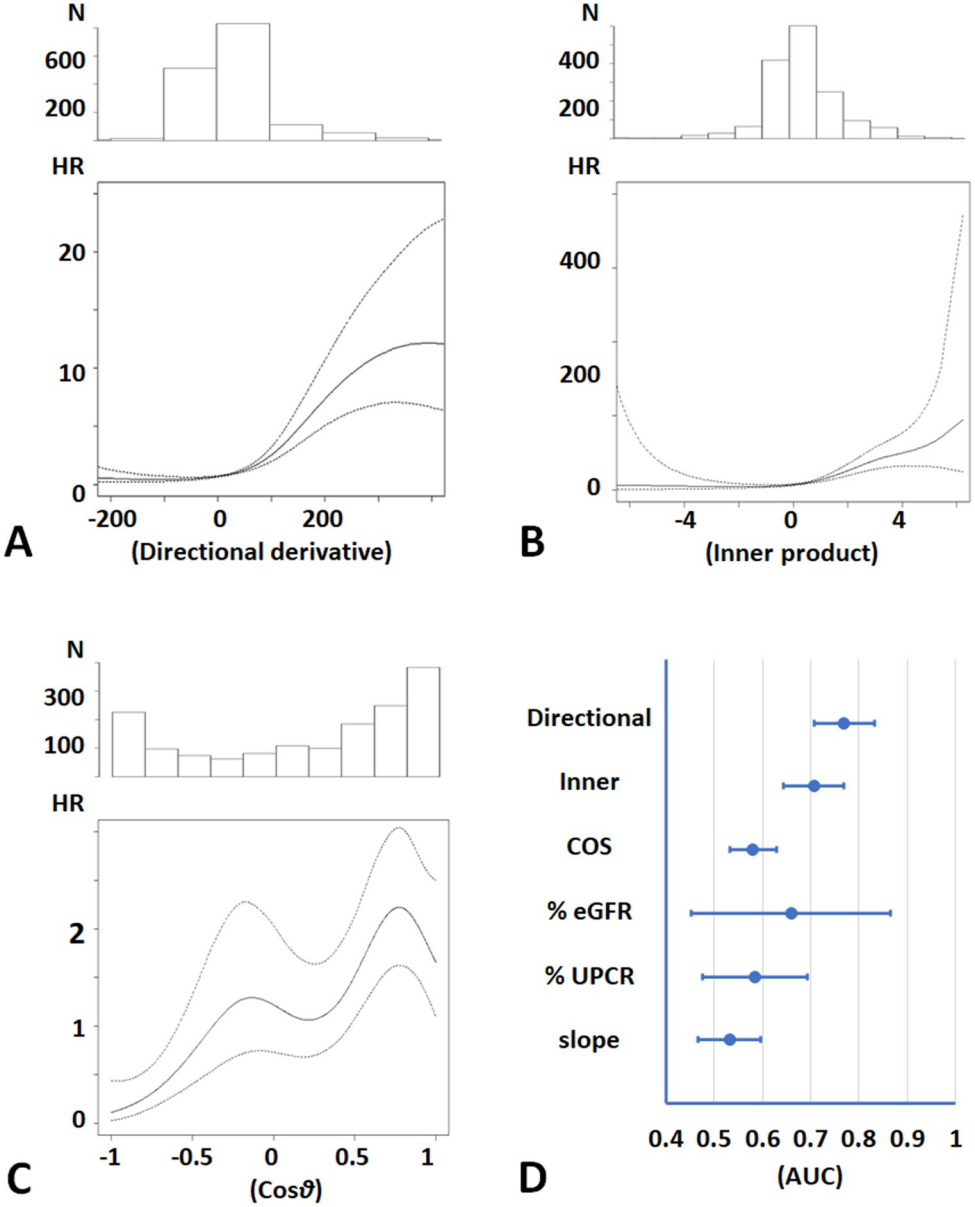
ESKD risk and indices of CKD progression. **A, B, C.** Histograms of the indices of CKD progression and the HR of ESKD are shown in the upper and lower panels, respectively. HRs were adjusted for the baseline characteristics. **A.** Directional derivative. **B.** Inner product. **C.** Cos*θ*. **D.** AUCs for the prediction of ESKD. AUCs of Directional, Inner, and Cos*θ* were adjusted for the baseline characteristics. AUCs of % eGFR, % UPCR, and slope were adjusted for the baseline characteristics with eGFR and UPCR. The AUCs are as follows: the directional derivative, 0.77 (95% CI 0.71, 0.83); the inner product, 0.71 (95% CI 0.64, 0.77); Cos*θ*, 0.58 (95% CI 0.53, 0.63); % eGFR change, 0.66 (95% CI 0.45, 0.87); % UPCR, 0.58 (95% CI 0.48, 0.69); eGFR slope, 0.53 (95% CI 0.47, 0.60). Abbreviations: Directional, the directional derivative; Inner, the inner product; COS, Cos*θ*; % eGFR, % eGFR change; % UPCR, % UPCR change; slope, eGFR slope: HR, hazard ratio adjusted for baseline characteristics; AUC, area under the receiver operating characteristic curve adjusted for baseline characteristics.

The directional derivative, the inner product, and Cos*θ* were positively associated with ESKD risk (*p*<0.0001, for each index) (Fig 4A to 4C). Positive values of the directional derivative and the inner product were associated with high ESKD risk and indicated an accelerated CKD progression with increasing directional derivative and inner product (Fig 4A and 4B). Adjusted HRs of less than 1 for ESKD risk corresponded to negative values of Cos*θ*, and adjusted HRs of more than 1 corresponded to positive values of Cos*θ* (Fig 4C).

Adjusted AUCs of the directional derivative, the inner product, and Cos*θ* were statistically significantly higher than 0.5 (*p*<0.05, for each index) (Fig 4D). On the other hand, % changes in eGFR and UPCR as well as eGFR slope did not show sufficient accuracy for diagnosing high-risk patients. Their AUCs were lower than that of the directional derivative (*p*<0.0001, for each index).

#### Subclass analysis

The accuracies of the prediction of ESKD events were compared among the indices in DM, non-DM, old, and young groups (S2 Fig). Comparison of the accuracies of *r*, *z*, eGFR, and UPCR in every group showed that *z* had the highest accuracy, and the accuracy of UPCR was lower than those of others. The AUC of *z* was larger than that of eGFR in the DM, old, and young groups (*p*<0.0001, for each group), and no significant difference was among the indices observed in the non-DM group (*p* = 0.37).

Among the indices of CKD progression, the directional derivative and inner product showed higher accuracies for the progression of ESKD than other indices in any subgroup (S2 Fig). The AUCs of the directional derivative and the inner product were consistently statistically significantly higher than 0.5.

## Discussion

This study was the first to use vector analysis to elucidate the pathophysiology of CKD. We mathematically transformed the CKD stage and developed a completely new model for ESKD risk estimation. The CKD-stage vector field has unique and convenient characteristics, which have never been reported before to the best of our knowledge [10,11]. Treating CKD stage as a coordinate made it clear that the distance from CKD stage G2 A1 to a patient’s data exponentially reflects ESKD risk. Considering applications to clinical settings, a logarithmic chart for the estimation of ESKD risk was developed. This enables a very easy estimation of ESKD risk only by measuring the length from CKD stage G2 A1 to a patient’s data using a ruler. Moreover, the risk ratio of ESKD can be easily estimated. This chart will be a good tool for therapies and patient education.

The directional derivative as the change rate in ESKD risk more accurately predicts events than the eGFR slope. This is due to the ingenious structure of the directional derivative, which includes eGFR, proteinuria, and their slopes. First, models including both eGFR and proteinuria can more accurately predict outcome events than models including either eGFR or proteinuria [19–21]. Second, eGFR does not always indicate ESKD itself, because it is calculated on the basis of serum creatinine level. Kanda et al. and Matsushita et al. showed a u-shaped relationship between % eGFR change and ESKD risk [9,22]. Kovesdy et al. also reported that an increase in eGFR slope is associated with ESKD risk [23]. One of the reasons why the increase in eGFR does not always mean an improvement in kidney function is that an increase in eGFR can be observed when muscle mass decreases and malnutrition occurs. The directional derivative is calculated on the basis of time-course data similar to eGFR slope and can be used as a surrogate endpoint for clinical research investigating treatment effects and risk factors.

The inner product and Cos*θ* provide a new concept that the direction of the highest ESKD risk exists for each patient and sensitively reflects the influence of CKD on its progression. In our study, the inner product and Cos*θ* showed that most of the patients in CKD stage G3b or later proceed in the direction of the highest ESKD risk. eGFR trajectory studies have shown that there are several patterns of eGFR change and also presented the trend that most of the patients in CKD stage G3b or later show a decrease in eGFR [24–28]. These lines of evidence suggest that recovery of kidney function is very difficult after CKD stage G3b. Furthermore, a decrease in eGFR shows several nonlinear patterns, and there are many cases where a decrease in eGFR does not reflect ESKD risk [29]. For example, when both eGFR and proteinuria decrease in a patient with early diabetic nephropathy, it is difficult to judge whether CKD is progressing or not [30]. In such a case, indices directly reflecting ESKD risk are needed to accurately monitor ESKD risk. This study’s indices will play an important role as a guide for monitoring kidney function and planning therapies.

Our models are applicable to clinical settings in the context of their limitations. First, the CKD patient data in Japan were used. Future studies using data from patients with various characteristics, such as diseases, medication, and countries, are required to validate the CKD-stage vector field. Second, CKD is a risk factor for CVD and death. It will be medically beneficial to show the CKD-stage vector field in relation to CVD and mortality. Third, the patients in this study were mainly in CKD stage G2 or later. It will take many years for sufficient ESKD events to occur in patients with high eGFR in CKD stage G1. Therefore, further studies of such patients should be conducted [9]. Fourth, we proposed one model. To select this model, we considered various mathematical theories and variables such as the complex plane, not only the vector field, on the basis of which, we created various models. Subsequently, we selected the model that was in line with previous medical evidence and was as easy to calculate as possible. In future studies, there is a possibility of developing a mathematically and clinically superior model. Fifth, in this study, we were unable to perform the time-course analysis of various data related to CKD, such as potassium and phosphorus, and prescribed medicine, this will be our next research topic. However, the vector field enabled the use of advanced mathematical models such as dynamical systems, chaos, and state space models, which have never been used in nephrology research before [18]. By analyzing these models together with conventional statistical models, one may be able to obtain new and interesting results.

In conclusion, this study showed that the transformed CKD stage treated as a vector field enables the easy and accurate estimation of ESKD risk and will be useful in clinical treatments and studies. Additionally, the application of vector analysis, commonly used in mathematics, neuroscience, and machine learning, is suggested for clinical studies of CKD and other diseases, thereby expanding the boundaries of research in this field.

## Materials and methods

### Study design and ethics

In this study, the CKD-stage vector fields were validated using data from CKD patients who visited Kawasaki Medical School Hospital from January 1st, 2014 to December 31st, 2020 (Kawasaki CKD cohort study). The data was extracted from the electronic medical record database in Kawasaki Medical School Hospital on July 8th, 2023. This retrospective cohort study was approved by the Kawasaki Medical University and Hospital Ethics Committee (No. 5306–01, 6047–00). The exemption for informed consent from participants is also approved by the Kawasaki Medical University and Hospital Ethics Committee. The study was performed in accordance with the relevant guidelines and the Declaration of Helsinki. We did not have access to information that could identify individual participants during or after data collection.

CKD patients who regularly visited the outpatient clinic of Kawasaki Medical School were registered (n = 3,714) (S3 Fig). The patients whose eGFR was 75 mL/min/1.73 m^2^ or less and whose eGFR and proteinuria were measured at least twice were included in this study. The exclusion criteria were as follows: patients younger than 20 years, patients on any type of dialysis, and patients who had malignancies. Renal transplant patients were not included in this study, although renal transplantation is not one of the exclusion criteria. The first-visit data of a patient were treated as baseline data. A total of 1,564 patients were included in the analysis and followed up for three years.

### Assumption

In this study, we hypothesized that the distance from CKD stage G2 A1, which was defined as the origin of the Euclidean space, to a patient’s data on eGFR and proteinuria (*r*) is associated with ESKD risk. Then, we assessed using the CKD cohort data whether mathematical models of the exponential function of *r* (CKD-stage vector field) reflect ESKD risk and investigated whether change-over-time of the model indicates an increase in risk.

### Variables

The variables were CKD-related factors and medications (S4 Table) [4,5,31–33]. Baseline characteristic variables were age; gender; diabetes mellitus; hypertension; history of CVD; serum albumin, sodium, potassium, calcium, phosphorus, low-density lipoprotein, and uric acid levels; white blood cell count; hemoglobin level; and use of renin-angiotensin-aldosterone system (RAAS) inhibitors, statins, and erythropoietin-stimulating agents. eGFR was calculated using the equation for the Japanese population on the basis of serum creatinine level measured using the enzymatic method [34]. There were no missing data on these variables. The primary outcome was ESKD. If no outcomes were observed within the follow-up period, the observation data were treated as censored data. The onset of ESKD was defined as the initiation of renal replacement therapy. None of the patients in this study had received a kidney transplantation.

The time-series data obtained on medical examination days at least one month between examinations were used in the calculation of changes in eGFR and UPCR. Percent (%) changes in eGFR and UPCR per year were calculated on the basis of the baseline and last eGFR and UPCR measured. On the basis of time-series data on eGFR, eGFR slope (mL/min/1.73 m^2^/year) was calculated using mixed models [35–37]. Briefly, the model of eGFR slope took the form

$$eGFR\left(t\right)=\;b0+\;b0_{i}+\left(b1\;+\;b1_{i}\right)*t+\;e_{i}$$ (2)

where *i* indicates a patient; *t* is time; *b*0 and *b*1 are respectively the fixed intercept and slope; *e*_*i*_ is an error; and *b*0_*i*_ and *b*1_*i*_ are the random intercept and slope, respectively. Percent changes in eGFR and UPCR per year (% eGFR change, % UPCR change, %/year) were calculated on the basis of the baseline and the last eGFR and UPCR measured as follows:

$$\%\;eGFR\;change=\;\frac{\left(last\;eGFR-baseline\;eGFR\right)}{baseline\;eGFR}*\frac{1}{period}*100$$ (3)

where period refers to the follow-up period [9]. % UPCR change was calculated similarly.

### Coordinate transformation

CKD stage is to some extent difficult to mathematically analyze. First, the difference in the units of eGFR and proteinuria prevented the use of eGFR and proteinuria as mathematical variables for equivalent analysis. Second, the starting points of eGFR and proteinuria differ; e.g., with CKD progression, proteinuria increases from 0, whereas eGFR decreases from a high eGFR.

On the basis of the fact that ESKD risk increases with the worsening of CKD by one degree for each of CKD stages G and A, the coordinate transformation was conducted (S4 Fig). CKD stage G progression by one degree corresponds to a 15 mL/min/1.73 m^2^ decrease in eGFR. Then, the National Kidney Foundation and the US Food and Drug Administration have evaluated the eGFR slope in early CKD for a hypothetical population with a mean eGFR of 75 mL/min/1.73 m^2^ [7,35]. Thus, the coordinate is the Euclidean space, whose origin is CKD stage G2 A1. Therefore, eGFR was transformed into a new eGFR (*x*) as follows.


$$x=\frac{75-eGFR}{15}$$ (4)

The cutoff levels for CKD stage A are UPCRs of 0.15 and 0.5 g/gCr. Then, UPCR increases 10/3 times with the worsening of CKD for CKD stage A. In this study, the minimum UPCR was treated as 0.045 g/gCr, and UPCR was defined as

$$UPCR=0.045*{\left(\frac{10}{3}\right)}^{y}$$ (5)

Then, UPCR was converted to a new UPCR (*y*) as follows.


$$y=\frac{\text{ln}\left(UPCR\right)+3.1}{1.2}$$ (6)

The eGFR and UPCR at all visits were converted to x and y, respectively. Here, the units of *x* and *y* are dimensionless and represent one degree of CKD stages G and A. On the basis of these transformations, CKD stage was transformed into Cartesian coordinates. The units of *x* and *y* were named “stage”. The distance from the origin to a patient’s data was measured as *r*:

$$r=\sqrt{x^{2}+y^{2}}$$ (7)

Moreover, on the basis of the time-series data of *x* and *y*, the slopes of *x* and *y* were calculated using mixed models similarly to eGFR slope as described above [35].

### CKD-stage vector field in scalar field

In previous studies, ESKD risks are usually investigated mainly using logistic regression and Cox proportion hazards models. The characteristic of methods using these models is that the odds ratio (OR) and HR are calculated using the exponential function of Napier’s constant, *e*: OR = exp (coefficient). Thus, the exponential function was used for a candidate CKD-stage vector field of *φ*:

$$\begin{array}{ll} \emptyset \left(x,\;y\right) & =\text{exp}\left(C\sqrt{x^{2}+y^{2}}\right)\\ & =\text{exp}\left(Cr\right) \end{array}$$ (8)

where *C* is a constant and *φ*(0, 0) = 1 is the reference.

When the ESKD risk at a certain CKD stage is known, *C* can be estimated using the distance from a reference CKD stage to that CKD stage. For example, the distance from CKD stage G2 A1 as the reference to CKD stage G3 A2 is between 1 G stage and 1 A stage, that is, $\sqrt{1+1}=1.4$ stage. Then, given that the HR in CKD stage G3A2 is 2.72,

$$C=\frac{\text{ln}\left(HR\right)}{r}=\frac{\text{ln}\left(2.72\right)}{1.4}=0.71$$ (9)

Large-scale cohort studies showing ESKD risk on the basis of CKD stage were searched using PubMed and manually. A literature search of PubMed was conducted for studies from January 2015 to December 2021. Key terms related to “chronic kidney disease”, “GFR”, “proteinuria”, “cohort”, and “dialysis” were included. Three reports, whose sample sizes are larger than about 5,000, were included in the analysis [13,16,17]. By applying data from the meta-analysis of three cohort studies to the calculation, we obtained data on *C*s. The mean and 95% confidence interval (CI) of *C* were estimated using the bootstrap method with random sampling 1,000 times on the basis of the data from the three large-scale cohort studies [13,16,17]. Then, it was assessed whether the CKD-stage vector field reflects ESKD risk using CKD cohort data.

### Logarithmic chart and its use

The logarithmic chart was developed on the basis of eGFR and UPCR. The axis of eGFR is from 75 mL/min/1.73 m^2^, and its scale span is 15 mL/min/1.73 m^2^/cm. UPCR is the logarithmic axis, and scales are given to frequently referred values. In the chart, CKD stage G or A is shown in the same length (1 cm) when this file is printed on A4 paper.

Generally speaking, potential can be mathematically evaluated at the two points *x*_1_ and *x*_2_ to obtain the work that a force (vector) performs over any trajectory between these two points. Percent changes between two-point-measured data on eGFR and proteinuria have been shown as surrogate endpoints of ESKD, which have characteristics similar to potential [6,9]. Thus, the change in ESKD risk between the previous and present medical examinations can be easily estimated without considering the trajectory.

Given that the lengths of previous and latest data from the origin are *r*_1_ and *r*_2_, respectively, the risk ratio of ESKD is as follows:

$$\begin{array}{ll} risk\;ratio & =\;\frac{\text{exp}\left(r_{2}\right)}{\text{exp}\left(r_{1}\right)}\\ & =\text{exp}\left(r_{2}-r_{1}\right) \end{array}$$ (10)

Thus, risk estimation and comparison can be conducted using a ruler. For example, suppose a patient’s first-time measurement of *r*_1_ is one stage. A doctor may say to the patient that your ESKD risk is 2.7 times higher than that at CKD stage G2 A1. If the next measurement of *r*_2_ is three stages, the doctor will say that your ESKD risk has become 7.4 times higher than before and shows the need for a thorough examination. By using the logarithmic chart this way, it is very useful for the diagnosis of CKD severity, evaluation of the therapeutic effects, and therapy planning.

### CKD progression

Let *z* = ∅(*x*, *y*) where *x* and *y* are functions of time (*t*). Then, CKD progression can be estimated using the total derivative of *z* as.

$$\begin{array}{ll} \frac{dz}{dt} & =\frac{\partial z}{\partial x}\frac{dx}{dt}+\frac{\partial z}{\partial y}\frac{dy}{dt}\\ & =\frac{C\text{exp}\left(Cr\right)}{r}\left(x\frac{dx}{dt}+y\frac{dy}{dt}\right)\\ & =\frac{C\text{exp}\left(Cr\right)}{r}\left(x,\;y\right)\left(\begin{array}{c} \frac{dx}{dt}\\ \frac{dy}{dt} \end{array}\right) \end{array}$$ (11)

$\frac{dx}{dt}$ and $\frac{dy}{dt}$ are approximately represented as slopes of *x* and *y*, respectively. The vector at the baseline (*x*, *y*) indicates the direction of the highest increment in ESKD risk for each patient (direction of highest ESKD risk) (S5 Fig). Here, the slopes of x and y were calculated on the basis of the mixed model similarly to the eGFR slope [35]. Then, the slopes of *x* and *y* are substituted for $\left(\frac{dx}{dt},\;\frac{dy}{dt}\right)$ in the equation.

$\frac{dz}{dt}$ is the directional derivative according to the direction of CKD progression in a patient (direction of the effect of CKD) (S5 Fig). Then, the inner product is calculated as (x,y)dxdtdydt. In physics, the inner product shows the work that a force (vector) performs on an object to displace it, so the inner product is considered to indicate the influence of CKD [38]. Where *θ* is the angle between the direction of the highest ESKD risk and the direction of the effect of CKD, Cos*θ* can also be calculated. Cos*θ* shows the direction of CKD progression compared with the direction of the highest ESKD risk.

### Statistical analyses

Variables are presented as mean ± standard deviation (SD) or median (interquartile range). Statistical significance was defined as *p*<0.05. Intergroup comparisons of parameters were performed using the chi-square test, t-test, Mann-Whitney U test, one-way ANOVA, or Kruskal-Wallis test as appropriate.

The relationships between indices and the risk of ESKD were evaluated using Cox proportional hazards models. The multivariate Cox proportional hazards models with splines were adjusted for baseline characteristics such as age; gender; DM; hypertension; history of CVD; serum albumin, sodium, potassium, calcium, phosphorus, low-density lipoprotein, and uric acid levels; white blood cell count; hemoglobin level; and use of RAAS inhibitors, statins, and erythropoietin-stimulating agents with or without eGFR and UPCR as appropriate. The results are shown with an adjusted HR.

The ESKD risk in each CKD stage was estimated using the mean *z*. Next, the ESKD risk ratio in each CKD stage compared with that in CKD stage G2 A1was calculated in the Kawasaki CKD cohort study. Then, the estimated risks were compared with the risk ratios using Spearman’s correlation coefficient (*ρ*).

The ROC curve was used to evaluate the performance of an index to predict ESKD within three years. The AUC with 95% CI was estimated using the bootstrap method with random sampling 1,000 times. Because ROC is affected by the characteristics of the study population, AUCs were adjusted using generalized linear models with the baseline characteristic variables such as age; gender; DM; hypertension; history of CVD; serum albumin, sodium, potassium, calcium, phosphorus, low-density lipoprotein, and uric acid levels; white blood cell count; hemoglobin level; and use of RAAS inhibitors, statins, and erythropoietin-stimulating agents; with or without eGFR and UPCR as appropriate. AUCs were compared between indices using the bootstrap method. For the stratification analysis, the patients were categorized into DM, non-DM, young (less than 65 years), and old (65 years or older). For each group, the comparisons of AUCs between indices were also performed similarly.

These analyses were conducted using Mathematica 13.3 (Wolfram Research, Inc., Illinois, USA), SAS version 9.4 (SAS, Inc., Cary, North Carolina), STATA 18 (StataCorp LLC, Texas, USA), and R version 4.3.2 (The R Project for Statistical Computing, Vienna, Austria).

## Supporting information

S1 Fig Distribution of z. (PDF)

S2 Fig AUCs for the prediction of ESKD in subclasses. (PDF)

S3 Fig Study population. (PDF)

S4 Fig Coordinate transformation. (PDF)

S5 Fig Spatial relationship between patient data and CKD progression. (PDF)

S1 Table Baseline characteristics by stage. (PDF)

S2 Table ESKD events by stage. (PDF)

S3 Table Indices of CKD progression by stage. (PDF)

S4 Table Definition of variables. (PDF)
