# Supplementary material for: Mathematical expansion and clinical application of chronic kidney disease stage as vector field
Source: PLoS One. 2024 Mar 13;19(3):e0297389. doi: 10.1371/journal.pone.0297389 (PMC10936765; doi:10.1371/journal.pone.0297389)
Supplement: S1 Fig — (PDF) [file pone.0297389.s001.pdf]

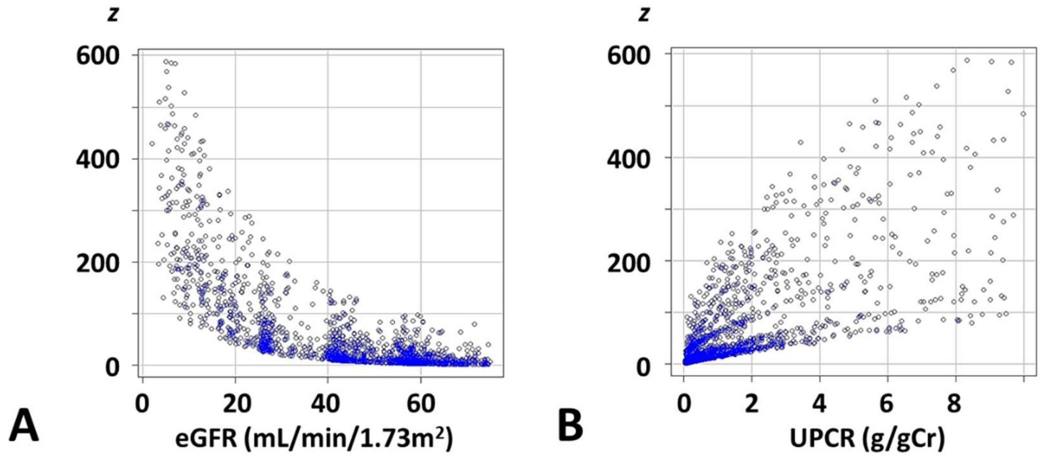

S1 Fig. Distribution of z.

A. eGFR and z.

B. UPCR and z.

Abbreviations: eGFR, estimated glomerular filtration rate; UPCR, urinary protein-to-creatinine ratio.
