# Supplementary material for: Mathematical expansion and clinical application of chronic kidney disease stage as vector field
Source: PLoS One. 2024 Mar 13;19(3):e0297389. doi: 10.1371/journal.pone.0297389 (PMC10936765; doi:10.1371/journal.pone.0297389)
Supplement: S2 Fig — (PDF) [file pone.0297389.s002.pdf]

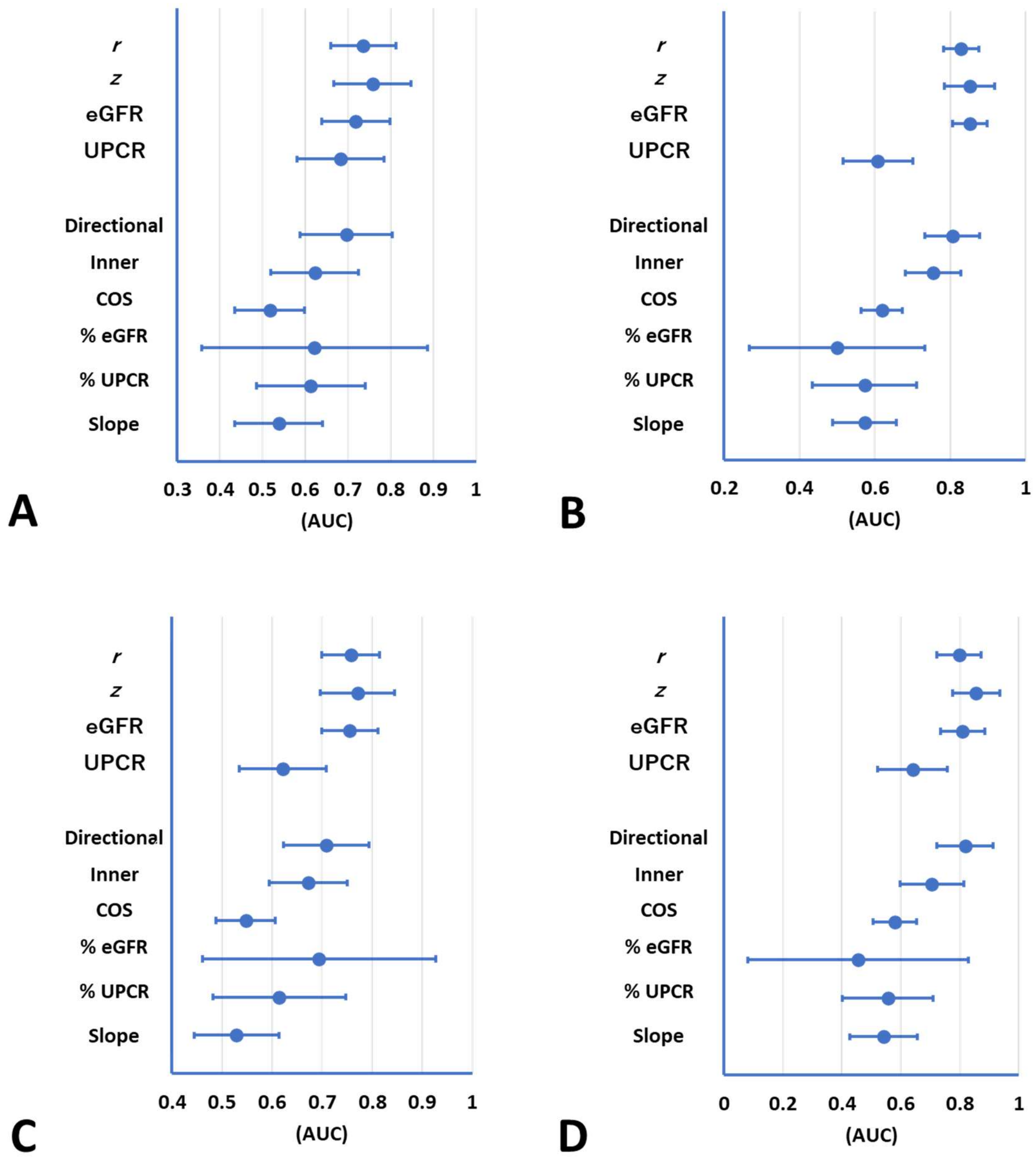

S2 Fig. AUCs for the prediction of ESKD in subclasses.

AUCs of *r*, *z*, Directional, Inner, and Cosθ were adjusted for the baseline characteristics (reference Methods). AUCs of eGFR and UPCR were adjusted for the baseline characteristics with UPCR and eGFR, respectively. AUCs of % eGFR, % UPCR, and slope were adjusted for the baseline characteristics with eGFR and UPCR.

A. DM patients

B. Non-DM patients.

C. Old patients (65 ≤ age).

D. Young patients (age < 65).

Abbreviations: AUC, area under the receiver operating characteristic curve adjusted for baseline characteristics; eGFR, estimated glomerular filtration rate; UPCR, urinary protein-to-creatinine ratio; Directional, the directional derivative; Inner, the inner product; COS, Cosθ; % eGFR, % eGFR change; % UPCR, % UPCR change; slope, eGFR slope; DM, diabetes mellitus.
