# Supplementary figures and images for: Mathematical expansion and clinical application of chronic kidney disease stage as vector field
Source: PLoS One. 2024 Mar 13;19(3):e0297389. doi: 10.1371/journal.pone.0297389 (PMC10936765; doi:10.1371/journal.pone.0297389)

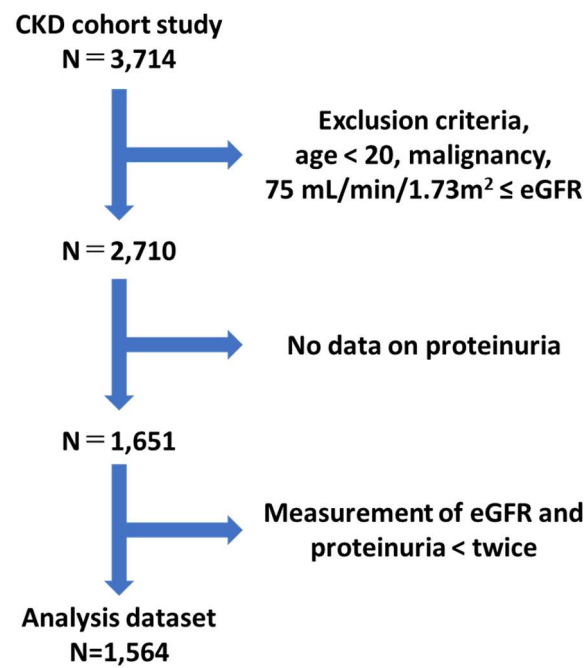

**S3 Fig. Study population.**

Abbreviation: eGFR, estimated glomerular filtration rate.

Supplement: S3 Fig — (PDF) [file pone.0297389.s003.pdf]
