# Supplementary material for: Mathematical expansion and clinical application of chronic kidney disease stage as vector field
Source: PLoS One. 2024 Mar 13;19(3):e0297389. doi: 10.1371/journal.pone.0297389 (PMC10936765; doi:10.1371/journal.pone.0297389)
Supplement: S5 Fig — (PDF) [file pone.0297389.s005.pdf]

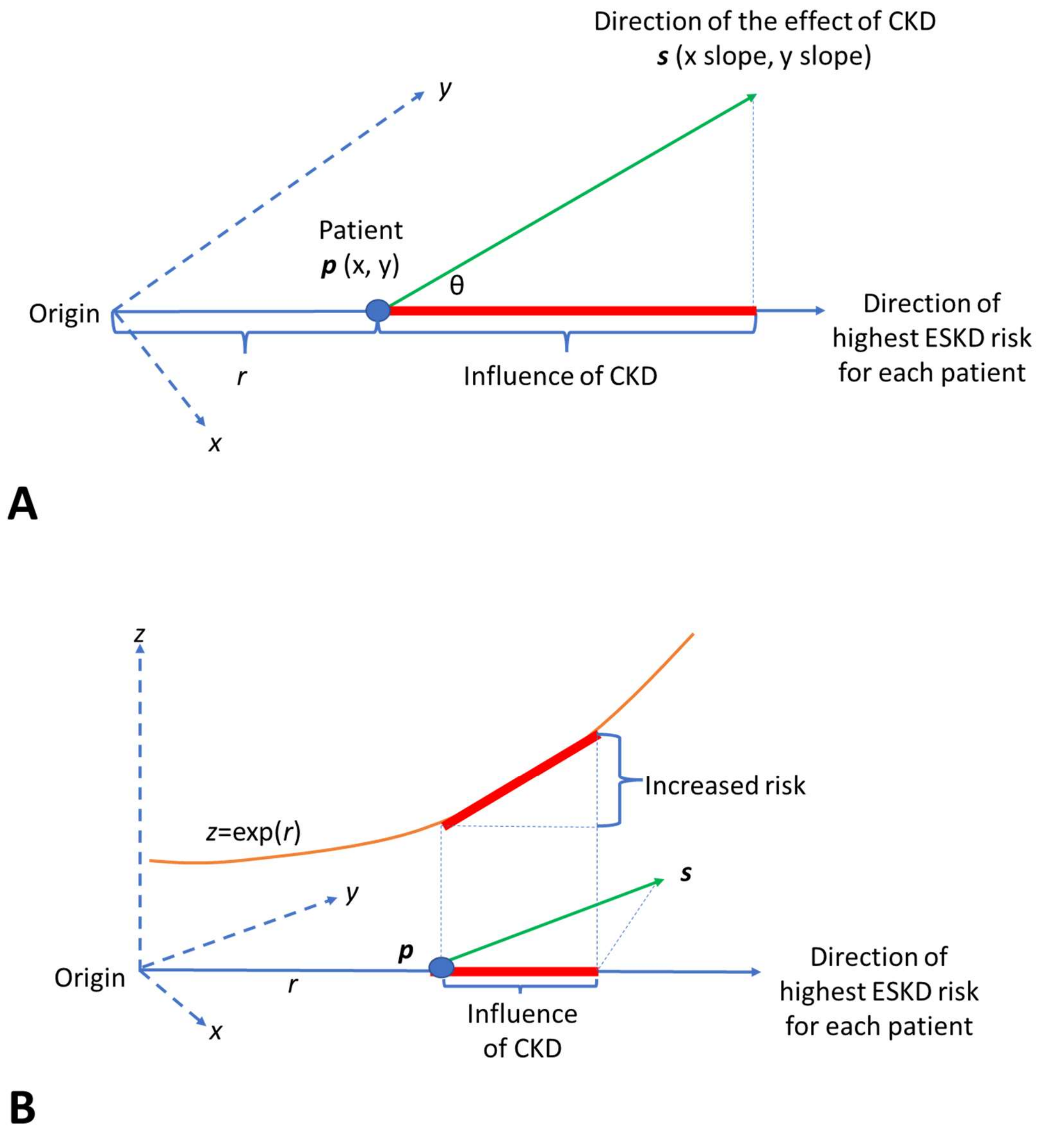

S5 Fig. Spatial relationship between patient data and CKD progression.

A. The direction from the origin to a patient's data (x, y) indicates the direction to which ESKD risk most rapidly increases. The direction of CKD progression of a patient (change in kidney function) is determined by the slopes of x and y (green line). Given that the angle between the highest ESKD risk line and the green line is θ, the influence of CKD progression on ESKD risk is shown as the inner product (red line).

B. x, y, and z show a three-dimensional relationship. The influence of CKD progression on ESKD risk is converted to a change in z.

Abbreviations: CKD, chronic kidney disease; ESKD, end-stage kidney disease.
