# Supplementary material for: Mathematical expansion and clinical application of chronic kidney disease stage as vector field
Source: PLoS One. 2024 Mar 13;19(3):e0297389. doi: 10.1371/journal.pone.0297389 (PMC10936765; doi:10.1371/journal.pone.0297389)
Supplement: S1 Table — (PDF) [file pone.0297389.s006.pdf]

S1 Table. Baseline characteristics by stage.

Distance group (stage)	All	Group 1 (0≦ to <1)	Group 2 (1≦ to <2)	Group 3 (2≦ to <3)	Group 4 (3≦ to <4)	Group 5 (4≦ to <5)	Group 6 (5≦)	*p* value
No. (%) with data	1564	60 (3.8)	260 (16.6)	375 (24.0)	357 (22.8)	288 (18.4)	224 (14.3)	
R, mean (SD), /stage	3.29±1.42	0.71±0.22	1.51±0.28	2.49±0.28	3.50±0.28	4.49±0.30)	5.52 (0.37)	<0.0001
Age, mean (SD), years	63.9±15.3	44.1±11.6	58.7±14.5	63.4±14.5	65.9±14.8	67.8±13.8	67.65±15.10	<0.0001
Male (%) with data	947 (60.5)	53 (88.3)	213 (81.9)	173 (46.1)	224 (62.7)	156 (54.2)	128 (57.1)	<0.0001
Comorbidity								
DM (%) with data	452 (28.9)	9 (15.0)	43 (16.5)	87 (23.2)	124 (34.7)	103 (35.8)	86 (38.4)	<0.0001
Hypertension (%) with data	1151 (73.6)	25 (41.7)	169 (65.0)	258 (68.8)	279 (78.2)	236 (81.9)	184 (82.1)	<0.0001
CVD (%) with data	206 (13.2)	1 (1.7)	14 (5.4)	30 (8.0)	51 (14.3)	54 (18.8)	56 (25.0)	<0.0001
Laboratory data								
eGFR, mean (SD), (mL/min/1.73m^2)	41.6±18.8	67.4±4.2	59.2±5.4	50.8±9.7	42.6±13.5	29.1±14.2	13.33±7.22	<0.0001
CKD stage G2 (%) with data	259 (16.6)	60 (100)	95 (36.5)	62 (16.5)	34 (9.5)	8 (2.8)	0 (0)	<0.0001
CKD stage G3a	472 (30.2)	0 (0)	165 (63.5)	153 (40.8)	123 (34.5)	31 (10.8)	0 (0)	

(%) with data								
CKD stage G3b (%) with data	326 (20.8)	0 (0)	0 (0)	160 (42.7)	100 (28)	58 (20.1)	8 (3.6)	
CKD stage G4 (%) with data	315 (20.1)	0 (0)	0 (0)	0 (0)	100 (28)	153 (53.1)	62 (27.7)	
CKD stage G5 (%) with data	192 (12.3)	0 (0)	0 (0)	0 (0)	0 (0)	38 (13.2)	154 (68.8)	
x	2.23±1.26	0.51±0.28	1.05±0.36	1.62±0.65	2.16±0.90	3.06±0.95	4.11±0.48	<0.0001
Albumin, mean (SD), g/dL	3.8±0.6	4.4±0.6	4.1±0.5	4.0±0.5	3.8±0.6	3.6±0.7	3.44±0.65	<0.0001
Sodium, mean (SD), mmol/L	140.4±3.1	141.3±1.9	140.7±2.5	140.8±3.0	140.4±3.3	134.0±3.3	139.44±3.36	<0.0001
Potassium, mean (SD), mmol/L	4.5±0.7	4.2±0.5	4.3±0.6	4.3±0.5	4.4±0.6	4.7±0.7	4.81±0.77	<0.001
Calcium, mean (SD), mg/dL	8.9±0.6	9.1±0.5	9.1±0.5	9.1±0.5	9.0±0.6	8.8±0.7	8.47±0.76	<0.0001
Phosphorus, mean (SD), mg/dL	3.6±0.9	3.2±0.8	3.2±0.5	3.4±0.6	3.3±0.6	3.8±0.8	4.81±0.77	<0.0001
LDL, mean (SD), mg/dL	107.7±33.1	112.9±37.0	107.4±29.1	107.9±29.7	109.0±35.2	110.6±37.3	100.33±32.03	0.015
Uric acid, mean (SD), mg/dL	6.4±1.7	5.8±1.2	5.8±1.3	6.0±1.5	6.4±1.5	6.9±2.0	7.11±1.82	<0.0001
WBC, mean (SD),	6.8±4.1	6.5±1.8	6.6±2.0	6.9±6.6	6.9±2.4	7.1±3.8	6.73±2.98	0.67

10^3/μL								
Hemoglobin, mean (SD), g/dL	12.7±2.4	15.0±1.8	14.0±1.8	13.2±2.0	12.8±2.3	11.9±2.2	10.43±1.78	<0.0001
UPCR, median (interquartile range), g/gCre	0.67 [0.21, 1.95]	0.07 [0.05, 0.09]	0.14 [0.09, 0.23]	0.37 [0.17, 0.62]	1.14 [0.47, 1.95]	1.71 [0.93, 4.04]	3.84 [2.08, 6.06]	<0.0001
CKD stage A1 (%) with data	297 (19.0)	60 (100)	141 (54.2)	74 (19.7)	20 (5.6)	2 (0.7)	0 (0)	<0.0001
CKD stage A2 (%) with data	389 (24.9)	0 (0)	119 (45.8)	169 (45.1)	74 (20.7)	27 (9.4)	0 (0)	
CKD stage A3 (%) with data	878 (56.1)	0 (0)	0 (0)	132 (35.2)	263 (73.7)	259 (89.9)	224 (100)	
y	2.22±1.18	0.33±0.33	0.94±0.50	1.66±0.70	2.49±0.82	3.06±0.81	3.62±0.56	<0.0001
z	67.2±97.05	2.08±0.42	4.72±1.32	12.52±3.56	34.47±9.66	93.31±28.27	267.27±106.25	<0.0001
Medication								
RAASI (%) with data	638 (40.8)	15 (25.0)	101 (38.8)	161 (42.9)	164 (45.9)	130 (45.1)	67 (29.9)	<0.0001
Statin (%) with data	579 (37.0)	20 (33.3)	77 (29.6)	144 (38.4)	144 (40.3)	122 (42.4)	72 (32.1)	0.014
ESA (%) with data	408 (26.1)	0 (0.0)	13 (5.0)	46 (12.3)	78 (21.8)	136 (47.2)	135 (60.3)	<0.0001

Continuous variables are shown as mean ± SD or median [interquartile range] as appropriate. Categorical variables are shown as n (%).

Abbreviations: DM, diabetes mellitus; CVD, cardiovascular disease; eGFR, estimated glomerular filtration rate; CKD, chronic kidney disease; LDL, low-density lipoprotein; WBC, white blood cell; UPCR, urinary protein-to-creatinine ratio; RAASI, renin-angiotensin-aldosterone system inhibitor; ESA, erythropoietin-stimulating agent
