# Supplementary material for: Mathematical expansion and clinical application of chronic kidney disease stage as vector field
Source: PLoS One. 2024 Mar 13;19(3):e0297389. doi: 10.1371/journal.pone.0297389 (PMC10936765; doi:10.1371/journal.pone.0297389)
Supplement: S2 Table — (PDF) [file pone.0297389.s007.pdf]

S2 Table. ESKD events by stage.

Distance group (stage)	All	Group 1 (0≦ to <1)	Group 2 (1≦ to <2)	Group 3 (2≦ to <3)	Group 4 (3≦ to <4)	Group 5 (4≦ to <5)	Group 6 (5≦)	*p* value
N (%) with data	179 (11.4)	0 (0.0)	1 (0.4)	1 (0.3)	9 (2.5)	50 (17.4)	118 (52.7)	<0.0001
Follow up period (days)	818.5±389.4, 1095 (490, 1095)	982.3±260.5, 1095 (1095, 1095)	993.7±248.1, 1095 (1095, 1095)	915.1±330.2, 1095 (888.5, 1095)	843.7±371.9, 1095 (532, 1095)	792.4±387.5, 1083.5 (507.5, 1095)	403.2±377.8, 286.5 (70, 666.5)	<0.0001

Categorical variables are shown as n (%).
Follow-up period is shown as mean ± SD and median [interquartile range].
Abbreviation: ESKD, end-stage kidney disease.
