# Supplementary material for: Mathematical expansion and clinical application of chronic kidney disease stage as vector field
Source: PLoS One. 2024 Mar 13;19(3):e0297389. doi: 10.1371/journal.pone.0297389 (PMC10936765; doi:10.1371/journal.pone.0297389)
Supplement: S3 Table — (PDF) [file pone.0297389.s008.pdf]

S3 Table. Indices of CKD progression by stage

Distance group (stage)	All	Group 1 (0≦ to <1)	Group 2 (1≦ to <2)	Group 3 (2≦ to <3)	Group 4 (3≦ to <4)	Group 5 (4≦ to <5)	Group 6 (5≦)	*p* value
Directional derivative, mean (SD)	29.08±70	-0.57±1.04	0.03±3.51	0.13±10.42	5.13±26.46	47.78±69	142.42±107.15	<0.0001
Inner product, mean (SD)	0.45±1.46	-0.1±0.18	0.01±0.53	-0.01±1.04	0.22±1.32	0.94±1.92	1.58±1.68	<0.0001
Cosθ, mean (SD)	0.43 [-0.43, 0.79]	-0.51 [-0.90, -0.03]	0.05 [-0.76, 0.73]	0.17 [-0.66, 0.71]	0.31 [-0.47, 0.79]	0.68 [0.25, 0.88]	0.66 [0.45, 0.81]	<0.0001
% eGFR change, mean (SD), %/year	-4.8±15.6	-1.19±12.14	-2.33±11.58	-0.46±14.3	-4.32±14.7	-10.1±17.38	-12.86±18.93	<0.0001
% UPCR change, mean (SD), %/year	5.33±65.67	7.62±58.75	6.18±62.29	9.4±63.98	2.59±60.54	10.28±67.42	-7.38±79.4	0.12
eGFR slope, mean (SD), mL/min/1.73 m^2/year	-2.86±5.7	2.41±5.27	-0.87±5.36	-1.32±5.18	-2.6±5.95	-5.24±5.13	-6.47±3.87	<0.0001

Continuous variables are shown as mean ± SD or median (interquartile range) as appropriate.

Abbreviations: eGFR, estimated glomerular filtration rate.
