# Supplementary material for: Mathematical expansion and clinical application of chronic kidney disease stage as vector field
Source: PLoS One. 2024 Mar 13;19(3):e0297389. doi: 10.1371/journal.pone.0297389 (PMC10936765; doi:10.1371/journal.pone.0297389)
Supplement: S4 Table — (PDF) [file pone.0297389.s009.pdf]

S4 Table. Definition of variables.

Variable	Definition	Unit
Age	Continuous variable: Age	years
Male	Binomial variable: Male	0, 1
DM	Binomial variable: Diabetes mellitus	0, 1
HT	Binomial variable: Hypertension	0, 1
CVD	Binomial variable: Cardiovascular disease	0, 1
eGFR	Continuous variable: eGFR	mL/min/1.73 m^2
Alb	Continuous variable: Albumin level	g/dL
Na	Continuous variable: Sodium level	mmol/L
K	Continuous variable: Potassium level	mmol/L
Ca	Continuous variable: Calcium level	mg/dL
IP	Continuous variable: Phosphorus level	mg/dL
LDL	Continuous variable: LDL level	mg/dL
UA	Continuous variable: Uric acid level	mg/dL
WBC	Continuous variable: WBC	10^3/μL
Hb	Continuous variable: Hemoglobin level	g/dL
UPCR	Continuous variable: UPCR level	g/gCre
RAASI	Binomial variable: Use of RAASI	0, 1
Statin	Binomial variable: Use of statin	0, 1
ESA	Binomial variable: Use of ESA	0, 1

Abbreviations: DM, diabetes mellitus; HT, hypertension; CVD, cardiovascular disease; eGFR, estimated glomerular filtration rate; LDL, low-density lipoprotein; UA, uric acid; WBC, white blood cell; UPCR, urinary protein-to-creatinine ratio; RAASI, renin-angiotensin-aldosterone system inhibitor; ESA, erythropoietin-stimulating agent.
